# Phase separation in flowering control: DCP5 and SSF co-regulate *FLC* by liquid-liquid phase separation

**DOI:** 10.1093/plcell/koad163

**Published:** 2023-06-07

**Authors:** Ching Chan

**Affiliations:** Assistant Features Editor, The Plant Cell, American Society of Plant Biologists, Rockville, MD, USA; Department of Life Science, National Taiwan Normal University, Taipei 11677, Taiwan

The floral transition, the switch from vegetative to reproductive development, represents an integrated output downstream of multiple environmental stimuli and intracellular signaling pathways in plants. *FLOWERING LOCUS C* (*FLC*) is one of the best-characterized targets delaying flowering (Boss et al. 2004). By associating *FLC* expression levels among a panel of Arabidopsis natural accessions, Wang et al. (2020) identified *SISTER of FCA* (*SSF*) as a naturally polymorphic regulator. SSF directly binds to the *FLC* promoter and regulates *FLC* transcription. To further explore the mechanism of SSF function, **Wanyi Wang and colleagues** (Wang et al. 2023) surveyed SSF interacting proteins and identified DECAPPING 5 (DCP5), a component of the processing body. SSF and DCP5 play opposite roles in regulating *FLC* transcription and hence flowering time.

Phase separation is a process by which biomolecules de-mix from the cellular environment independent of the membrane and assemble into biomolecular condensates. Super-resolution confocal microscopy revealed the characteristic spot-like spherical pattern of biomolecular condensates for both SSF-GFP and DCP5-GFP expressed in transgenic Arabidopsis and *Nicotiana benthamiana*. The fluorescence signals were recovered after photobleaching, indicating that the dense phase contains dynamic molecules that can be exchanged within the condensate and/or with the surroundings. Similarly, SSF-GFP and DCP5-mCherry recombinant proteins displayed liquid droplet patterns and the capability to recover from photobleaching. Moreover, the droplets of recombinant proteins can fuse together to form larger droplets—another characteristic of liquid-liquid phase separation (Laflamme and Mekhail 2020).

A common phenomenon in the protein composition of these membraneless compartments is the prevalence of low-complexity sequence domains or intrinsically disordered regions (Cuevas-Velazquez and Dinneny 2018). Low-complexity sequence domains/intrinsically disordered regions are protein regions with an over-representation of polar and nonstructuring residues. These regions provide a high degree of conformational flexibility and increase the accessibility for protein-protein interactions and the feasibility for post-translational modification. Prion-like domains (PrDs), which display disordered properties, were annotated in both SSF and DCP5. Deletion of the PrDs abolished SSF-GFP and DCP5-mCherry phase separation and the SSF-DCP5 interaction in planta. Complementation of *ssf* or *dcp5* mutants by the corresponding deletion construct (SSFΔPrD/DCP5ΔPrD) did not rescue the mutant phenotypes (early flowering and reduced *FLC* transcript levels in *ssf* and delay flowering along with elevated *FLC* transcript levels in *dcp5*). Therefore, the PrD domains are critical for SSF and DCP5 function and phase separation. Interestingly, overexpression of DCP5 promoted SSF phase separation in Arabidopsis and *N. benthamiana* but not vice versa. Overall, the authors proposed that SSF acts as a protein scaffold to anchor on the *FLC* locus. This scaffold facilitates the interaction of other transcriptional regulators (such as DCP5) to fine-tune *FLC* expression (see Fig.).

**Figure.**
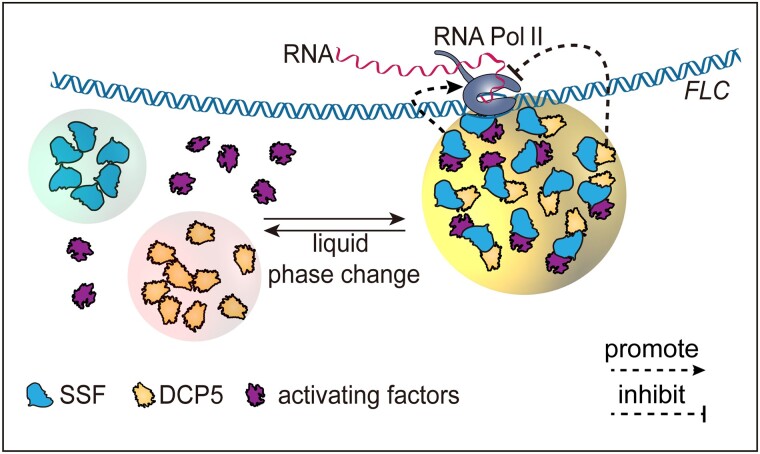
SSF and DCP5 co-regulate *FLC* expression in flowering control. SSF and DCP5 form liquid droplets independently. SSF directly binds *FLC* chromatin and recruits RNA Pol II to promote *FLC* expression. SSF acts as a protein scaffold on *FLC* to recruit other regulatory factors. The SSF-DCP5 interaction inhibits *FLC* expression. Adapted from Wang et al. (2023) Figure 10.

The functional significance of biomolecular condensates highlights the complexity of intracellular organization beyond membrane-bound organelles. The independent assembly and disassembly processes at the right place and the right time in response to various environmental signals are intriguing. Further studies using large-scale proteomics and advanced imaging techniques are anticipated to further delineate the biomolecular properties of liquid-liquid phase separation in various plant developmental and physiological processes.
